# A Major *Diplotaxis harra*-Derived Bioflavonoid Glycoside as a Protective Agent against Chemically Induced Neurotoxicity and Parkinson’s Models; In Silico Target Prediction; and Biphasic HPTLC-Based Quantification

**DOI:** 10.3390/plants11050648

**Published:** 2022-02-27

**Authors:** Atallah F. Ahmed, Zhi-Hong Wen, Ahmed H. Bakheit, Omer A. Basudan, Hazem A. Ghabbour, Abdullah Al-Ahmari, Chien-Wei Feng

**Affiliations:** 1 Department of Pharmacognosy, College of Pharmacy, King Saud University, P.O. Box 2457, Riyadh 11451, Saudi Arabia; basudan@ksu.edu.sa (O.A.B.); 438105699@student.ksu.edu.sa (A.A.-A.); 2 Department of Pharmacognosy, Faculty of Pharmacy, Mansoura University, El-Mansoura 35516, Egypt; 3 Department of Marine Biotechnology and Resources, National Sun Yat-sen University, Kaohsiung 804, Taiwan; wzh@mail.nsysu.edu.tw; 4 Department of Pharmaceutical Chemistry, College of Pharmacy, King Saud University, P.O. Box 2457, Riyadh 11451, Saudi Arabia; abakheit@ksu.edu.sa; 5 Department of Medicinal Chemistry, Faculty of Pharmacy, University of Mansoura, Mansoura 35516, Egypt; ghabbourh@yahoo.com; 6 Department of Obstetrics and Gynecology, Kaohsiung Medical University Hospital, Kaohsiung 807377, Taiwan; 1080532@kmuh.org.tw; 7 Center for Cancer Research, Kaohsiung Medical University, Kaohsiung 807377, Taiwan

**Keywords:** *Diplotaxis harra*, anti-inflammatory, neuroprotective, anti-Parkinson agent, HPTLC, isorhamnetin-3-O-β-D-glucoside, in silico prediction

## Abstract

Oxidative stress and chronic inflammation have a role in developing neurodegenerative diseases such as Parkinson’s disease (PD) and inflammatory movement disorders such as rheumatoid arthritis that affect millions of populations. In searching for antioxidant and anti-inflammatory molecules from natural sources that can counteract neurodegenerative diseases and arthritis, the flavonoid-rich extract of *Diplotaxis harra* (DHE) was selected based on its in vitro antioxidant and anti-inflammatory activities. DHE could inhibit the inducible nitric oxide synthase (iNOS) and cyclooxygenase-2 (COX-2) expressions in the lipopolysaccharide (LPS)-stimulated RAW 264.7 macrophages from 100% to the level of 28.51 ± 18.67 and 30.19 ± 5.00% at 20 μg/mL, respectively. A TLC bioautography of DHE fractions using 1,1-diphenyl-2-picryl-hydrazyl radical (DPPH) led to the isolation of a major antioxidant compound which was identified by X-ray diffraction analysis as isorhamnetin-3-O-β-D-glucoside (IR3G). IR3G also exhibited a potent anti-inflammatory activity, particularly by suppressing the upregulation of iNOS expression, similar to that of dexamethasone (DEX) at 10 μM to the level of 35.96 ± 7.80 and 29.34 ± 6.34%, respectively. Moreover, IR3G displayed a strong neuroprotectivity (>60% at 1.0^−4^–1.0^−3^ μM) against 6-hydroxydopamine (6-OHDA)-challenged SHSY5Y neuroblastoma, an in vitro model of dopaminergic neurons for Parkinson’s disease (PD) research. Accordingly, the in vivo anti-Parkinson potentiality was evaluated, where it was found that IR3G successfully reversed the 6-OHDA-induced locomotor deficit in a zebrafish model. A study of molecular docking and molecular dynamic (MD) simulation of IR3G and its aglycone isorhamnetin (IR) against human acetylcholine esterase (AChE), monoamine oxidase B (MAO-B), and Polo-like kinase-2 (PLK2) was performed and further outlined a putative mechanism in modulating neurodegenerative diseases such as PD. The free radical scavenging, anti-inflammatory through anti-iNOS and anti-COX-2 expression, and neuroprotective activities assessed in this study would present partial evidence for the potentiality of *D. harra*-derived IR3G as a promising natural therapeutic agent against neurodegenerative diseases and inflammatory arthritis. Finally, a biphasic HPTLC method was developed to estimate the biomarker IR3G in *D. harra* quantitatively.

## 1. Introduction

Research outcomes to date suggest the close linking between oxidative stress and chronic inflammation [1], which has a role in the development of inflammatory disorders such as neurodegenerative diseases such as Parkinson’s disease (PD) and Alzheimer’s disease (AD) [2], arthritis [3,4], and many other inflammation-based ailments. In addition, a relationship was found between neurodegeneration and the susceptibility to or the development of arthritis [5]. Therefore, several studies pointed out the pivotal protective role of natural antioxidants, anti-inflammatory, and neuroprotectants in combatting these pathological conditions. Many anti-inflammatory and neuroprotective drugs have been clinically examined for a variety of neurological and arthritic diseases, but to date, the results have been unsatisfactory or bearing intolerable side effects [6,7,8]. Consequently, the development of a more valued pharmacological strategy based on antioxidant and anti-inflammatory molecules from natural (food/plant) sources that may prevent or counteract neurodegenerative diseases and arthritic pain has been highly required. 

Since natural products and related derivatives represent over 50% of all drugs used clinically, the isolation of biologically active compounds from medicinal plants is highly interesting. Plants belonging to the mustard family (Brassicaceae, syn. Cruciferae) are distributed worldwide, and many of them have been traditionally used as anti-arthritic [9] and antirheumatic [10]. In this context, plants of genus *Diplotaxis* provided supporting evidence for this traditional use as they exhibited anti-inflammatory [11,12,13] and antioxidant/antiradical [13,14,15] activities since inflammation and oxidative stress are linked to inflammatory movement disorders, including rheumatoid arthritis [16,17,18]. Genus *Diplotaxis* includes edible and medicinal plants [12,19,20] of about 30 species, mainly distributed in Central Europe, the Mediterranean region [21], and Southwest Asia [22], including Saudi Arabia [23]. Phytochemical investigations on the herbs of genus *Diplotaxis*, including *Diplotaxis harra*, have been disclosed the presence of flavonoids [11,24,25,26,27], isothiocyanate-releasing glucosinolates [28,29], phenolic acids [19], and phytosterols [15]. Most of the isolated flavonoids belong to the mono- and di-glycosides of the flavonols isorhamnetin [24,26], then of quercetin and kaempferol [25,26], in addition to free isorhamnetin, quercetin, and apigenin aglycones [26,27]. *Diplotaxis harra* (Forsskal) Boissier is a short-lived perennial to an annual desert herb. It is locally known in Arabian countries as Harra (due to its pungent taste). A phenolic-rich organic fraction from the *D. harra* flowers demonstrated high antioxidant activity [19]. The alcoholic extracts and certain isolated flavonoids from the flowers and aerial parts exhibited cytotoxic activity against intestinal cancer [11], colon carcinoma, and many other cancer cell lines [25,26], along with antiviral activity against virus types A and O of the foot-and-mouth disease [26]. Moreover, extracts and different constituents of *D. harra* were found to possess antibacterial, antifungal [19,24,28,29], and anti-acetylcholinesterase [14] potentialities. 

The organic extract of *D. harra* (DHE) was found to possess a significant flavonoid/phenolic (F/P) ratio and showed considerable in vitro radical scavenging and anti-inflammatory activities. This prompted us to isolate a major antioxidant flavonoid, isorhamnetin-3-O-β-D-Glucoside (IR3G), from DHE. Bioassay data revealed the potent anti-inflammatory and neuroprotective activities of IR3G against the lipopolysaccharide (LPS)-induced inflammation in murine macrophage RAW 264.7 cells and against two Parkinson’s models [30,31,32] based on the 6-hydroxydopamine (6-OHDA)-treated human neuroblastoma (SH-SY5Y) cells and zebrafish, respectively. A further underlying mechanism of the isolated flavonoid in modulating PD was partially anticipated by performing in silico molecular docking and molecular dynamic (MD) simulation analysis against selected PD-related neurogenic targets such as human acetylcholine esterase (AChE), monoamine oxidase B (MAO-B), and Polo-like kinase-2 (PLK2). IR3G as a biological marker in *D. harra* was finally quantified by a software-controlled HPTLC system using normal phase (NP)- and reverse phase (RP)-HPTLC. 

## 2. Results

### 2.1. The Phytochemical and Antioxidant Evaluation of DHE, and Isolation of a Major Antioxidant Compound

#### 2.1.1. The Total Phenolic and Total Flavonoid Contents (TPC and TFC), and the Antioxidant Activity of DHE

DHE was found to have a significant TPC (measured as gallic acid equivalent (80.43 ± 0.01 mg GAE/g)) and TFC (measured as catechin equivalent (54.26 ± 0.03 mg CTE/g)). The flavonoid/phenolic (F/P) ratio was 0.67, indicating that the plant is rich in flavonoids. The 2,2-diphenyl-1-picrylhydrazyl (DPPH) and 2,2′-azino-bis[3-ethylbenzthiazoline-6-sulfonic acid] (ABTS) radical scavenging assays revealed that DHE possesses a significant antioxidant capacity of IC_50_ 203.7–247.4 μg/mL (Table 1).

#### 2.1.2. Isolation and Identification of IR3G

**Figure 1:**
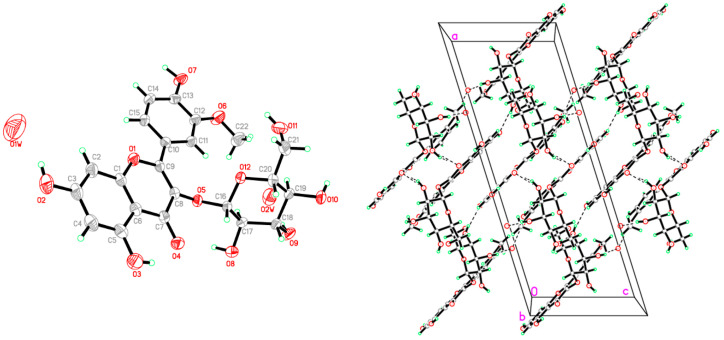
ORTEP diagram of IR3G. Displacement ellipsoids are plotted at the 40% probability level for non-H atoms (**left**). Molecular packing of IR3G with viewed hydrogen bonds is drawn as dashed lines (**right**).

A major UV-active constituent which exhibited a radical scavenging capacity, as visualized on thin-layer chromatography (TLC) combined with DPPH bioautography, was detected in the EtOAc fraction of DHE (Figure S1). Using reverse-phase (RP) C18 medium-pressure column chromatography enabled the isolation of a major pale-yellow crystalline compound. The molecular structure of the isolated compound was determined by a single-crystal X-ray diffraction analysis, using CuKa radiation, as isorhamnetin-3-O-β-D-glucoside (IR3G) (Figure 1, Figure S2 and Tables S1–S3). 

### 2.2. The Anti-Inflammatory, Neuroprotective, and Locomotion Deficit Inhibitory Activities

#### 2.2.1. The In Vitro Anti-Inflammatory Activity of DHE and IR3G on iNOS and COX-2 Expression

**Figure 2:**
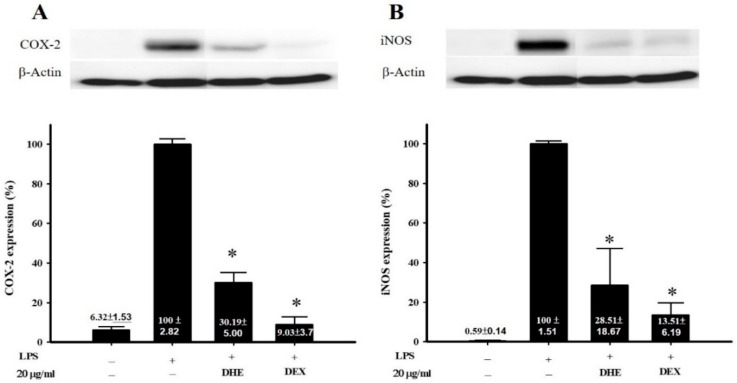
Anti-inflammatory activity of *Diplotaxis harra* extract (DHE) through inhibiting the expression of proinflammatory proteins COX-2 (**A**) and iNOS (**B**). Dexamethasone (DEX) was used as the positive. The values are mean ± S.E.M. (*n* = 6). *, Significantly different from LPS-activated group (*p* < 0.05).

The anti-inflammatory activity of DHE and IR3G against the expression of proinflammatory proteins (iNOS and COX-2) in the murine macrophage (RAW 264.7) cells stimulated with LPS was assessed according to previous studies [33,34]. It was found that DHE at 20 μg/mL concentration did not show cytotoxicity against the macrophage cells. However, DHE succeeded, at the same concentration, to significantly (*p* < 0.05) diminish the iNOS and COX-2 expression levels to 28.51 ± 6.19 and 30.19 ± 5.0%, relative to that (100% expression) of the control LPS only-treated cells, respectively (Figure 2).

**Figure 3:**
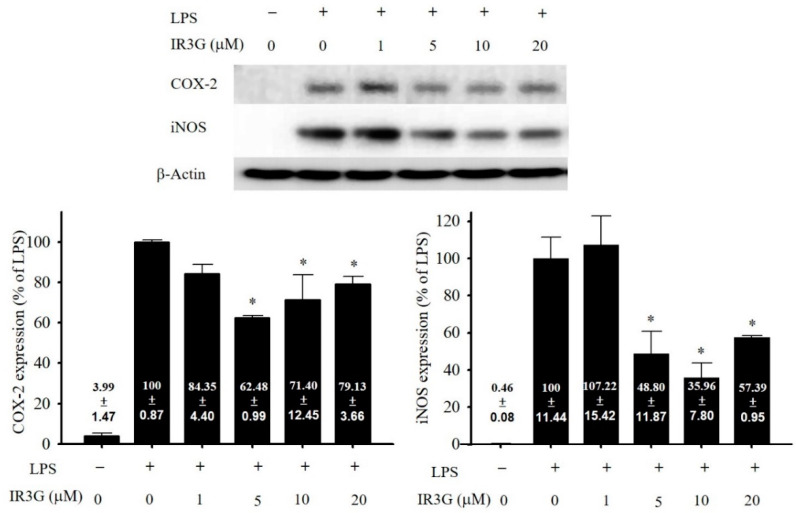
Anti-inflammatory activity of IR3G. Dexamethasone (DEX) was used as the positive control at 10 μM, inhibiting COX-2 and iNOS expressions from 100% in the LPS-stimulated cells to 15.97 ± 2.50% and 29.34 ± 6.34%, respectively. The values are mean ± S.E.M. (*n* = 6). *, Significantly different from LPS-activated group (*p* < 0.05).

The anti-inflammatory activity exhibited by DHE prompted us to estimate further the activity of its major flavonoid glucoside (IR3G) in different doses (1–20 μM). The results revealed that IR3G also displayed a considerable inhibitory effect against the LPS-upregulation of iNOS and COX-2 in macrophage cells at 5, 10, and 20 μM, but not at 1 μM. It downregulated the expression of the proinflammatory enzymes to the range of 57.39 ± 0.95 to 35.96 ± 7.80% for iNOS, and 79.13 ± 3.66 to 62.48 ± 0.99% for COX-2, relative to that showed (100%) by the LPS-only-stimulated cells. IR3G showed its highest inhibition level against iNOS (35.96 ± 7.80%) and COX-2 (62.48 ± 0.99%) expression at the doses of 10 and 5 μM, respectively (Figure 3). The level of iNOS inhibition obtained by IR3G was found to be nearly similar to that of dexamethasone (DEX, 29.34 ± 6.34%) at the same molar dose (10 μM).

#### 2.2.2. The Protective Effect of IR3G against the In Vitro 6-OHDA-Induced Neurotoxicity

**Figure 4:**
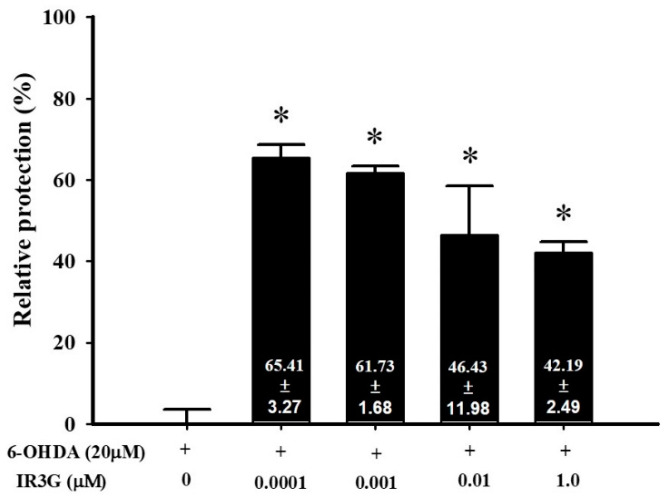
Neuroprotective effect of IR3G against 6-OHDA damage in SH-SY5Y cell. SH-SY5Y cells were pretreated with 0.0001, 0.001, 0.01, and 1μM IR3G for 1 h and then challenged with 20 μM 6-OHDA for 18 h. The relative protection level of the 6-OHDA-treated group was normalized to 0%. Our data showed that pretreatment of IR3G significantly rescued SH-SY5Y cells from 0 to nearly 50% of the relative protection rate. Data are presented as mean ± SEM, and each value represents the mean of three replicates and six samples. *, Significantly different from the 6-OHDA group. Relative neuroprotective activity of IR3G. Epigallocatechin gallate (EGCG) was used as the positive control at 1 μM, displaying relative neuroprotection of 29.97 ± 6.02%.

The in vitro neuroprotective activity of IR3G against the neurotoxicity induced by 6-OHDA in SHSY5Y neuroblastoma cells, human dopaminergic neurons frequently used in the study of PD [35], was achieved as previously described [36]. It was observed that the drastic effect of 6-OHDA on the SH-SY5Y dopaminergic neuroblastoma cells can be significantly reduced by pretreatment with IR3G at various concentrations (0.0001 to 1 μM) (Figure 4). A higher relative neuroprotective level of IR3G was achieved at a dose of 0.0001 μM (65.41 ± 3.27%). However, IR3G at a dose of 1 μM displayed more than 140% of the neuroprotective potency of epigallocatechin gallate (EGCG, 29.97 ± 6.02%) at the same molar concentration under the same conditions. These neuroprotectivity results prompted us to further explore the in vivo protective effect of IR3G against the 6-OHDA-induced locomotor deficit, Parkinsonian-like symptoms in the zebrafish model.

#### 2.2.3. The Protective Effect of IR3G on the In Vivo 6-OHDA-Induced Deficits in Locomotor Activity

**Figure 5:**
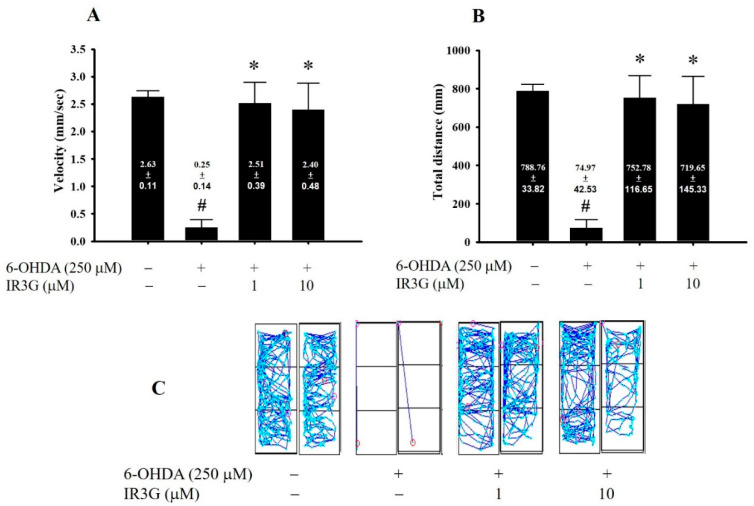
Effects of IR3G on the 6-OHDA-induced locomotor deficit in zebrafish PD model. Zebrafish larvae were pretreated with 1 or 10 μM IR3G from 9 h post-fertilization (hpf) to 5 days post-fertilization (dpf) and then challenged with 250 μM 6-OHDA from 2 to 5 dpf. (**A**) Mean velocity (mm/s); (**B**) Total swimming distance (mm); and (**C**) Typical swimming pattern in the control, 6-OHDA-challenged, 6-OHDA plus IR3G-treated groups are shown (*n* = 16). Data showed that IR3G significantly reversed the 6-OHDA-induced downregulation of locomotor activity at 1 or 10 μM. Each group contained 16 zebrafish, and data are represented as mean ± SEM. #, Significantly different from the control group (*p* < 0.05); *, Significantly different from the 6-OHDA group (*p* < 0.05).

Dopaminergic neuronal death usually results in mobility deficits. Zebrafish (*Danio rerio*) larva display a variety of complex undulatory swimming (locomotion) patterns which are controlled by the 300 neurons originating from the brain to the spinal cord [37]. In our experiment, zebrafish larva was treated with 250 μM 6-OHDA from the second to fifth-day post-fertilization (dpf). The locomotion activity was then assessed based on the total distance (in mm) traveled by fish in one 5 min session, the velocity of fish (mm/s), and the typical swimming pattern (Figure 5). The results clearly showed that exposure to 6-OHDA decreased the locomotor activity as manifested in decreasing the mean velocity (from 2.63 ± 0.11 to 0.25 ± 0.14 mm s^−1^) and the total swimming distance (from 788.76 ± 33.82 to 74.97 ± 42.53 mm) at 5 dpf (Figure 5A,B). The pattern of the active swimming was also lost in the 6-OHDA-exposed larva, contrary to the normal control (Figure 5C). Pretreatment with 1 μM IR3G significantly attenuated the 6-OHDA-induced locomotor deficit in zebrafish as represented by gaining nearly the same velocity (2.51 ± 0.39 mm s^−1^) and total swimming distance (752.78 ± 116.65 mm) as that of the non-intoxicated control. Furthermore, 10 μM IR3G also reversed velocity of the 6-OHDA-induced downregulation of mean velocity (from 0.25 ± 0.14 to 2.40 ± 0.48) and total swimming distance (from 74.97 ± 42.53 to 719.65 ± 145.33 mm). Moreover, the complexity of the pattern for the active swimming was nearly recovered to that of normal control by IR3G, more profoundly at 1 μM (Figure 5C).

### 2.3. In Silico Study

Generally, glucosides are the only glycosides absorbed from the small intestine, giving a higher plasma level [38]. Dietary flavonoid glycosides, including flavonol-3-O-glycosides, can also be hydrolyzed by human intestinal bacteria [39], releasing their corresponding aglycones. Furthermore, the genus Diplotaxis, including Diplotaxis harra, contains free flavonols such as isorhamnetin [26]. Therefore, we favored carrying out the molecular docking and dynamic simulation studies for the flavonol glucoside IR3G compared to its aglycone isorhamnetin (IR, Figure S2) to disclose their potential in neuroprotection and anti-Parkinson’s activity through interaction with three related target proteins.

#### 2.3.1. The Molecular Docking Study of IR3G and Its Aglycone IR

The docking was validated by the superimposition of the co-crystallized ligand onto the redocked ligand with the PDB structure target structure at root mean square deviation (RMSD) of <2 Å. The human acetylcholine esterase (AChE, PDB ID: 4M0E), monoamine oxidase B (MAO-B; PDB ID: 6FVZ), and Polo-like kinase-2 (PLK2; PDB ID: 4I5P) were selected as three possible pharmacological target proteins for IR3G and IR in the pathway of neuroprotective and anti-Parkinson’s activities.

**Figure 6:**
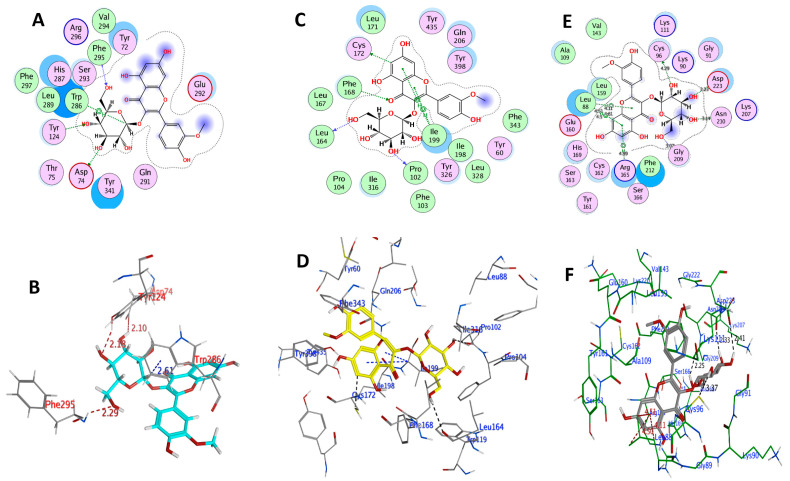
Docked conformation of IR3G within the binding pocket of AChE, MAO, and PLK2 as depicted in (**A**,**C**,**E**) for 2D docking poses; and in (**B**,**D**,**F**) for 3D docking poses, respectively.

**Figure 7:**
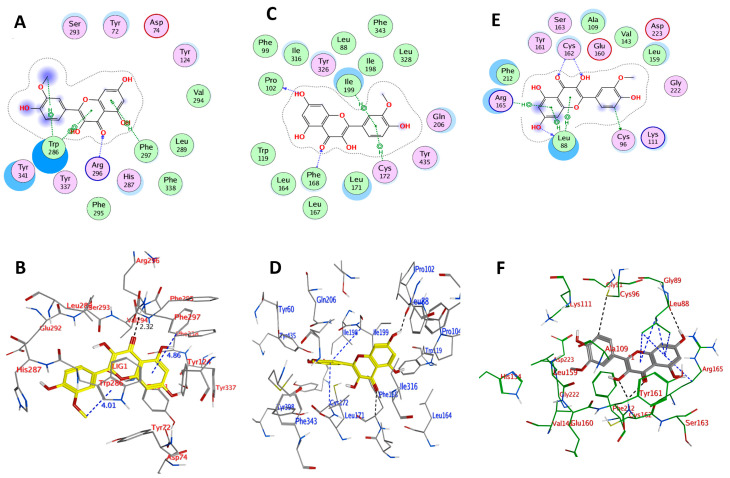
Docked conformation of IR within the binding pocket of AChE, MAO-B, and PLK2 as depicted in (**A**,**C**,**E**) for 2D docking poses; and in (**B**,**D**,**F**) for 3D docking poses, respectively.

AChE (PDB ID: 4M0E) has two identical chains (A, B). Previous studies found that the AChE inhibitors (AChEI) may interact with the positively charged Asp74 and Phe295 residues in the active site of AChE. The catalytic triad (side chains of His447, Glu334, and Ser203) is critical for AChE’s activity [40], and Phe338 is also involved in the interaction. Moreover, the residues Trp286, Trp86, Tyr341, Tyr337, and Tyr124 also contributed to strong ligand binding and activity [41]. When chain B was removed, the co-crystal selective inhibitor 1YL (Dihydrotanshinone I) was used to determine the active site. The amino acid residues Arg296, Asp74, Gln291, Glu292, Gly121, Gly342, His287, His447, Leu289, Phe295, Phe297, Phe338, Ser293, Trp286, Tyr72, Tyr124, Tyr337, Tyr341, Val294, and four water molecules were thus found in the binding pocket of the enzyme [42]. The results of docking IR3G and redocking of 1YL with 4M0E are summarized in Table 2 and Table S4, respectively, suggesting a relatively lower docking score (−6.4471 kcal mol^−1^) for 1YL relative to that of IR3G (−7.7045 kcal mol^−1^). Therefore, IR3G may strongly bind with AChE. In this context, we found that IR3G interacts with the critical amino acid residues Asp74, Phe295, Tyr124, and Trp286, among others (Table 2 and Figure 6). It forms a higher expected number of hydrogen bonds with critical residues than its aglycone IR (docking score −6.8919 kcal mol^−1^) which forms hydrogen bonds with Arg296, Phe297, and Trp286 (Table 2 and Figure 7).

A docking study was also performed for IR3G and IR against MAO-B (PDB ID: 6FVZ) to confirm the binding and orientations. The active site of the enzyme contains two hydrophobic cavities, the entry cavity (300 Å^3^), and the substrate cavity (400 Å^3^), which are separated by the Phe168, Leu171, Ile199, and Tyr326 side chains [43]. The aromatic cage is formed by two almost parallel tyrosyl residues (Tyr398 and Tyr435) and FAD [44]. Significantly, IR exhibited the same orientation of co-crystallized ligand, with the dihydroxy-phenyl ring at the 3,5,7-trihydroxy-4H-chromen-4-one terminal facing the entrance cavity and the aryl-substituted (4-hydroxy-3-methoxyphenyl) part facing the substrate cavity. Additional analysis of the ligand–protein complexes revealed that the IR3G and IR were bound to the large active site cavity created by the amino acid residues Gln206, Tyr326, Cys172, Ile316, Leu171, and Ile199 (Table 2 and Figure 6 and Figure 7). This demonstrated that both compounds have a considerable binding affinity with the MAO-B active site. Additionally, most inhibitors were maintained in the enzyme pocket via hydrogen bonds, hydrophobic, and pi–pi interactions. The docking scores of IR3G and IR were calculated as -5.863 and −7.529 kcal/mol, respectively, indicating a high binding capacity for the aglycone (IR) at the present docking pose (Figure 7). The IR interaction indicated a pi–pi interaction between the 2-phenyl ring against Ile199 and Cys172. Additionally, a hydrogen bond was identified between the 7-hydroxy and 3-oxo groups of chromen-4-one and Pro102 and Phe168, respectively.

The active site of PLK2 (PDB ID: 4I5P) is mainly composed of the amino acid residues Leu88, Cys96, Ala109, Lys111, Val143, Leu159, Glu160, Cys162, and Phe212 [45]. The binding energy and polar/nonpolar interactions were considered adequately in one docked pose of the most active compound IR3G. This docked pose was chosen from 100 conformations and possessed binding energy of −7.734 kcal/mol, generating 4H-bond interactions with the active site residues (Table 2 and Figure 6). The hinge region residues Asp223, Asn210, Gly209, and Cys96 created four H-bond interactions with four hydroxyl groups surrounding the glucopyranose ring of the ligand IR3G. Furthermore, IR3G interacts with Leu88 and Arg165 through pi–H. In addition, although IR interacts with the active site residues, its efficient docked pose demonstrated binding energy of −6.432 kcal/mol, indicating the capability of making 4H-bond interactions with the PLK2 hinge region residues (Table 2 and Figure 7). It has been observed that the interaction between the ligand and the hinge residue Cys172 is critical for binding. The binding patterns of IR3G and IR were found to be comparable to the binding patterns reported in the co-crystallized inhibitor of PLK2 in the study, supporting the selected pose of these compounds.

IR3G and IR were thus found to dock with negative binding energy, highlighting their potential binding affinity for the active sites of the PDB-linked targets (AChE, MAO-B, and PLK2). Based on the above findings, the selected docked conformations of IR3G and IR with the three target proteins were used in the following MD simulation study.

#### 2.3.2. MD Simulation Study of Protein–Ligand Complexes

MD simulation is a sophisticated computational method that can disclose the precise molecular interactions between the target protein and inhibitor at the atomic level [46]. To better evaluate the possible binding mode of IR3G or IR into AChE, MAO-B, and PLK2 binding sites as predicted by the docking study, MD simulation on the ligand–protein complexes was performed as follows. A completely solvated system containing explicit water molecules and ions was produced and optimized using energy minimization; the optimized system was then utilized as the input for a 20 ns MD protocol (see Materials and Methods). During the simulation, the root mean square deviation (RMSD) of the protein backbone and the heavy atoms of the ligand and root mean square fluctuations (RMSF) were used to analyze the MD data.

**Figure 8:**
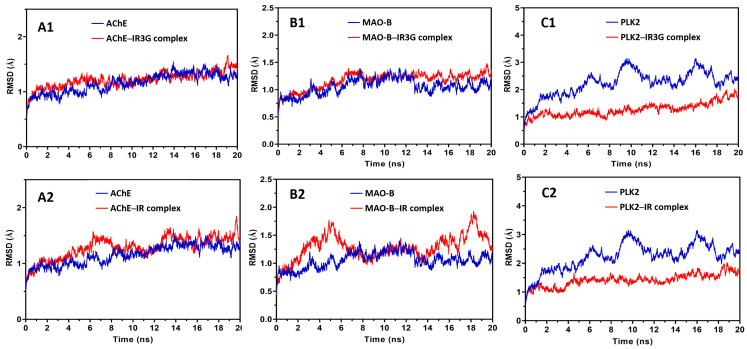
Online color molecular dynamic (MD) simulation analysis of isorhamnetin-3-O-β-D-glucoside (IR3G) with AChE (**A1**), MAO-B (**B1**), and PLK2 (**C1**) complexes and MD simulation analysis of isorhamnetin (IR) with AChE (**A2**), MAO-B (**B2**), and PLK2 (**C2**) complexes. The RMSD values of the protein backbone (blue) and protein–ligand complex (red) from their initial docking coordinates are presented.

As illustrated in Figure 8, the MAO-B-IR complex was considered stable, as both the protein structure and ligand-binding mode remained steady during the MD. An early increase in the protein backbone’s RMSD is noticed due to the loss of the position constraint on the protein carbons. However, the protein quickly approaches equilibrium, with the backbone RMSD fluctuating around 1.3 Å. The RMSD of the ligand positions indicates that IR already changes its binding mode in the first few steps of MD and achieves an equilibrium conformation with the protein after 6.5 ns of simulation. Additionally, the fluctuation of the complex began increasing again at 13.8 ns of simulation. It reached an equilibrium conformation at 19.8 ns, although the fluctuation of the complex remained less than 2.0 Å, indicating that the complex’s structure conformation was stable compared to the protein. Furthermore, the RMSD of the ligand positions indicates that IR3G already changes its binding mode in the few steps of MD and achieves an equilibrium conformation with the protein at most of the simulation (Figure 8). When comparing the RMSD values of IR3G and IR, we can conclude that the protein complex of MAO-B with IR3G is more stable than that of IR.

Figure 8 also showed that the AChE complexes with IR3G and IR were significantly stable, as both the protein structure and the ligand-binding mode were well preserved during the MD. After the first 8 ns of simulation, the RMSD of the protein backbone increases initially due to the absence of the position restraint on the protein carbons; however, the protein and complex quickly achieve an equilibrium within the following few ns, with a backbone RMSD fluctuating about 1.7 Å.

The complexes of PLK2-IR3G and PLK2-IR showed stability, as both the protein structure and the ligand-binding mode were well retained during the MD (Figure 8).

**Figure 9:**
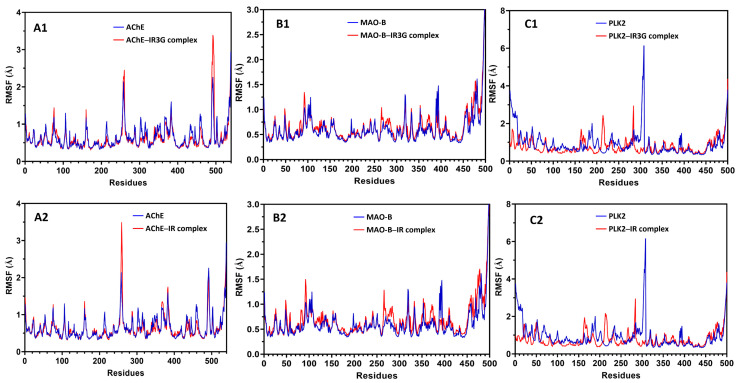
The root mean square fluctuation (RMSF) of the protein backbone during the MD simulation for isorhamnetin-3-O-β-D-glucoside (IR3G) complexes with AChE (**A1**), MAO-2 (**B1**), and PLK2 (**C1**) and RMSF of the protein backbone during the simulation for isorhamnetin (IR) complexes with AChE (**A2**), MAO-2 (**B2**), and PLK2 (**C2**).

Although the fluctuation of the protein backbone was <3.0 Å, the fluctuation of the complex was <1.5 Å, suggesting that the structural conformation of the complex was more stable than that of the protein. The stability of the target protein was further evaluated during the MD simulation by measuring the root mean squared fluctuation (RMSF) of the backbone atoms per residue (Figure 9). The results validated the enzyme’s strong stability during the MD, as an average RMSF value of 0.8 Å was achieved, and practically all residues had RMSF values less than 2.0 Å. Only the terminal protein residues exhibited more fluctuations in the complex of IR3G or IR with MAO-B, whereas in AChE-IR3G and AChE-IR complexes, the residues between 250–270 and 490–500 exhibited greater fluctuations. This was related to the gap between 258–265 and 494–497 residues, respectively, as seen in the protein structure (Figure 9). Moreover, in the PLK2-IR3G and PLK2-IR complexes, the residues between 158–174, 207–226, and 279–285 exhibited greater fluctuations, and the residues between 285–309 exhibited fewer fluctuations, as depicted in Figure 9, indicating that the complex’s structure conformation decreases the fluctuations of the active site residues when compared to the protein alone. The RMSD and RMSF analyses demonstrated the dependability of both the ligand–protein complex under investigation and the MD technique used to evaluate it.

### 2.4. HPTLC-Based Quantification of IR3G in DHE

**Figure 10:**
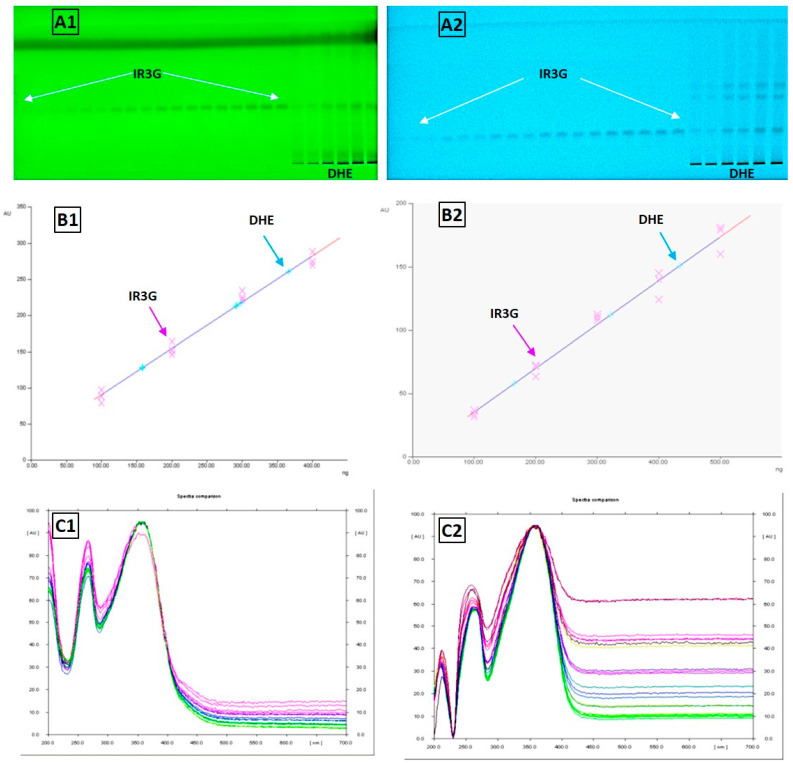
HPTLC analysis of IR3G and DHE. Chromatograms of IR3G and DHE detected on NP- (**A1**) and RP-HPTLC (**A2**) by UV 254 nm. Calibration plots based on UV absorbance at 254 nm against different concentrations (ng) of IR3G and DHE in NP- (**B1**) and RP-HPTLC (**B2**). Overlay UV spectra (λ 200–700 nm) of standard IR3G and IR3G in DHE, in NP- (**C1**) and RP-HPTLC (**C2**).

**Figure 11:**
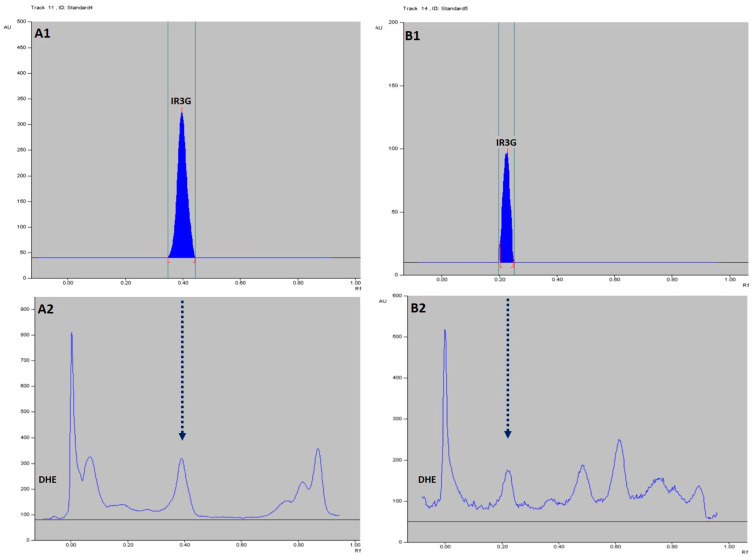
The chromatographic peaks of a standard IR3G and the extract DHE by NP- (**A1**,**A2**) and RP-HPTLC (**B1**,**B2**) at λ_max_ 254 nm. The *R_f_* values of IR3G (100–400/500 ng, each *n* = 3) in NP-HPTLC/EtOAc-CH_2_Cl_2_–HCOOH (9:1:1) = 0.398 ± 0.004 and in RP-HPTLC/CH_3_CN–H_2_O (3:7) = 0.222 ± 0.012.

The results of the HPTLC method used in the present study are summarized in Figure 10 and Figure 11, and Table 3. The isolated pure compound (IR3G) gave the typical UV spectrum of isorhamnetin-3-O-β-D-glucoside [47] and was further identified in the DHE sample tracks by the overlay UV spectra as demonstrated in Figure 10 and by the superimposed *R_f_* values in the NP- and RP-HPTLC systems (Figure 11). Further, IR3G produced linear calibration curves of absorbance versus concentration in the range of 100–400 ng/spot (r^2^ = 0.99296) in the NP-HPTLC method and the range of 100–500 ng/spot (r^2^ = 0.99154) in the RP-HPTLC method at λ_max_ 254 nm (Figure 10, Table 3). The peak areas correlated to the concentration of IR3G enabled the quantitative determination of this compound in DHE through the calibration curves. Therefore, it was found that 10 μL of DHE (0.3% *w*/*v* in water) was equivalent to 361.6 ng and 368.5 ng of IR3G as derived from the data of NP- and RP-HPTLC, respectively. Accordingly, IR3G is quantitatively estimated as 1.207–1.228% *w*/*w* in DHE or 0.1015–0.1034 *w*/*w*% in the dried herbal material of *D. harra*.

## 3. Discussion

Growing evidence shows that both inflammation and oxidative stress, associated with the increase of reactive oxygen species (ROS) and free radicals, can play, via many intermediate mechanisms, a critical role in the pathogenesis of neurodegenerative diseases such as Parkinson’s disease (PD) [48] and Alzheimer’s disease (AD) [49,50]; inflammatory movement disorders, including rheumatoid arthritis (RA) [16]; and other inflammatory diseases [51,52,53]. Moreover, it was found that neurodegeneration enhances the development of arthritis too [5]. In addition, the signs of PD were found more frequently in elderly RA cases when compared to healthy controls [54]. PD is a progressive neurodegenerative disorder mainly linked to the gradual loss of dopaminergic neurons in the midbrain *substantia nigra*, which results in tremors, postural instability, joint stiffness, bradykinesia, and other symptoms. PD is the second most widespread neurodegenerative disease after AD [55]. Several studies have already shown the involvement of proinflammatory proteins, such as iNOS and COX-2, particularly iNOS, in neurodegeneration [56,57,58,59] and arthritis [60,61,62]. Antioxidants that act as direct free radical scavengers or redox molecules are of great potential in combatting oxidative stress-related diseases with the associated inflammation signs and proinflammatory/oxidative damage biomarkers, including iNOS and COX-2 [16,63,64].

DHE proved a considerable antioxidant capacity in the present study, as shown by its DPPH and ABTS radical scavenging potency, correlated to the TPC and F/P ratio (Table 1). DHE could also significantly inhibit the upregulation in the expression of proinflammatory mediators iNOS and COX-2 in the LPS-activated murine macrophage cells from to the level of 30% at 20 μg/mL (Figure 2). The major isolated flavonoid glycoside (IR3G), in turn, revealed an antioxidant property (as shown by the DPPH bioautographic TLC; Figure S1) and displayed a significant anti-inflammatory activity in the LPS-induced inflammation in macrophage cells. However, a higher downregulation for iNOS expression was recorded for IR3G to the level of 35.96 ± 7.80% relative to COX-2 at 10 μM (Figure 3). The inhibition of COX-2 expression by DHE was found to be higher than that of IR3G, inferring the synergistic effect exerted by the phytochemical set, including total flavonoids, in the extract. This reducing potency in iNOS expression was in accordance with that demonstrated by other flavonoids [63,64], including flavonols such as isorhamnetin (IR), kaempferol, and quercetin [65].

Several studies have reported the critical role of natural antioxidants such as dietary flavonoids in modulating or decreasing the risk of PD [66,67], AD [68,69,70], and arthritis [71,72,73]. These flavonoid-related activities together with the biodata mentioned above for IR3G encouraged us to assess the neuroprotective effect of IR3G against chemically-induced neurotoxicity and locomotor lesion. The neuroprotectivity was evaluated against two Parkinson’s models [30,31,32], i.e., against the 6-OHDA-induced neurotoxicity in the human dopaminergic neuroblastoma SH-SY5Y cells and against the 6-OHDA-triggered locomotion insufficiency in zebrafish. The results of the present study clearly reflected a potent neuroprotectivity for IR3G at low concentrations (<0.1 μM) against the 6-OHDA-induced cellular neurotoxicity (Figure 4). An effect which was supported by a similar protectivity exerted by the 3′-O-demethylated derivative of IR3G (isoquercitrin) against the 6-OHDA-lesioned neuron-like pheochromocytoma (PC-12) cells [74]. Further, the in vitro neuroprotectivity of IR3G was echoed by the complete restoration of locomotor activity of zebrafish in the 6-OHDA-induced PD model at doses of 1 or 10 μM (Figure 5).

Numerous reports highlighted the neuroprotective mechanisms of flavonoids through inhibition of inflammatory mediators, free radical scavenging, suppressing lipid peroxidation, activation of endogenous antioxidant enzymes, inhibiting dopamine oxidation, and modulation of gene expression in neuronal cells [57,75,76]. Similar to IR3G, natural flavonol derivatives such as quercetin (and its 3-O-glycoside rutin), kaempferol, and myricetin displayed neuroprotective effects in the in vitro and in vivo 6-OHDA-PD designed models [77,78,79] and other chemically-induced models of PD [80] through protection against dopamine depletion and oxidative stress; and by maintaining the resting membrane potential of neurons [77,81]. As per the structure–antioxidant relationship, the guaiacol and catechol moieties (as ring B of IR3G and IR; and of quercetin, respectively) gave high oxygen radical absorbance capacity (ORAC) values [82], an activity which was diminished by the presence of one hydroxyl in ring B. Furthermore, flavonols are found to be more highly active than the corresponding flavones due to the presence of the 3-OH. In addition, the 3-OH/4-keto and the 4-keto/5-OH functionalities in the flavonols showed greater complexation ability [83] with transition metal ions, such as iron and copper, a phenomenon which is considered as the key mechanism of their biological activities, including radical scavenging [84].

Although the exact mechanism underlying the PD progression is uncertain, oxidative stress, inflammation, and mitochondrial dysfunction are believed to play significant roles [85]. Nevertheless, since flavonoids were capable of distributing widely in rat tissues, including the brain, and traveling across the blood–brain barrier [86,87], they can demonstrate antioxidant and anti-inflammatory effects and other possible pharmacodynamic interactions in the brain to combat the progress of PD. Therefore, flavonoids may further act by binding to the active site of PD-related enzymes in the brain. DHE and IR3G exerted antioxidant, anti-inflammatory, and neuroprotective effects in the present study, which could be partially beneficial in treating PD. However, computer-aided molecular docking and MD simulation studies were employed further to explore possible pharmacological targets for IR3G or IR, which may take part in modulating PD pathogenesis. The interactions of IR3G or IR with human AChE, MAO-B, and PLK2 were thus evaluated compared to the corresponding PDB-coded ligands (Table 2 and Table S4).

MAO inhibition is considered an important strategy for controlling neurodegenerative disorders, including PD, AD, and depression, by protecting dopamine from catabolism. The results of in vitro studies suggested that flavonoids can inhibit both MAO isoforms with IC_50_ values of μM to nM range. Furthermore, the docking studies and compiling SAR indicated that di-substitution at ring B enhances selectivity towards hMAO-B while the unsaturation of chromone ring is crucial for MAO inhibition [88]. These requirements are fulfilled in the structures of IR3G of DHE and IR molecules, although the substitution by 3-OH and 3-O-glucopyranose might be the reason for the difference in docking energy/binding affinity of IR (−7.529 kcal/mol) and IR3G (–5.863 kcal/mol) with hMAO-B, respectively (Table 2), with other protein–ligand interactions. However, MD simulation indicated that the protein complex of MAO-B with IR3G is more stable than that of IR as reflected from their RMSD values (Figure 8).

PLK2 plays a critical role in the phosphorylation of α-synuclein in the central nervous system, a modification that leads to the formation of pathologic aggregates (Lewy bodies) and, consequently, neurotoxicity, as in the case of PD and dementia [89]. Therefore, selective inhibition of PLK2 can mitigate the formation of phosphorylated α-synuclein and can be thus employed to treat PD. The present in silico study revealed a significant binding affinity for IR3G and IR with PLK2, with a superior interaction for IR3G (Table 2). The MD simulation proved considerably stable PLK2-IR3G and PLK2-IR complexes since protein structure and the ligand-binding mode were well retained in the definite time. It is worth mentioning that some flavonols showed an inhibitory effect against α-synuclein fibril formation [90], supporting the possible role of IR3G and IR in treating PD.

Moreover, data of docking study with AChE depicted that IR3G and IR showed higher docking scores than that of reference PDB ligand (Table 2 and Table S4). Similar interactions of other related flavonols, such as quercetin and kaempferol, with AChE could also disrupt the catalytic triad (His−Ser−Glu) of AChE [91]. The complexes of AChE with IR3G and IR were found to be significantly stable, as both the binding mode of the protein structure and the ligand were well preserved during the MD simulation specified time. The predicted in silico anti-AChE activity of IR3G and IR could be supported by the reported AChE inhibitory effect of different extracts and flavonol derivatives of *D. simplex* and *D. harra* [14]. Therefore, IR3G and IR might be considered as possible candidates to ameliorate PD, particularly the cognitive deficit or dementia in PD patients, via modulation of AChE [92].

In summary, the flavonoid-rich DHE exhibited significant antioxidant and anti-inflammatory activities, from which the isolated antioxidant glucoside IR3G also demonstrated anti-inflammatory action in addition to neuroprotective activities. The in vitro neuroprotectivity of IR3G was correlated to its potency against the in vivo Parkinson-like locomotion deficit model. Additionally, the protein–ligand docking and interaction profiling studies could predict the potential inhibitory effect of the DHE-derived IR3G/IR on AChE, MAO-B, and PLK2, which could improve neurodegenerative diseases, including PD. The efficiency of *D. harra* extract as anti-inflammatory, neuroprotective, and anti-Parkinson’s activity might be improved through the preparation of a defatted flavonoid-rich extract standardized by the activity-linked biomarker IR3G.

## 4. Materials and Methods

### 4.1. Plant Material

*D. harra* was collected in December 2013 from Western Riyadh, Saudi Arabia. The plant was identified by Dr. Mohammed Al-Yahya, Department of Pharmacognosy, College of Pharmacy, King Saud University. A voucher sample (# NSA016-1) was deposited at the Department of Pharmacognosy.

### 4.2. Extraction and Isolation of IR3G

The powdered aerial part of *D. harra* (2.5 kg) was extracted with 95% EtOH to give crude extract as a dark brown gum (210.6 g, DHE). A part of the extract (175.3 g) was mixed with H_2_O and filtered. The purified (precipitate-free) extract (141.8 g) was concentrated and then partitioned with *n*-hexane, CHCl_3_, EtOAc, and *n*-BuOH (saturated with H_2_O) to yield *n*-hexane (F1, 0.2 g), CHCl_3_ (F2, 4.6 g), EtOAc (F3, 4.1 g), and *n*-BuOH (F4, 48.6 g) fractions, respectively. A bioautographic method was employed to detect the antioxidant-rich fractions, using silica gel TLC and methanolic DPPH (0.5% *w*/*v*) as a visualizing spray reagent. F3, which showed a major antioxidant band at Rf = 0.54 (Si gel TLC; EtOAc–MeOH–H_2_O, 10:1.5:1), was selected for further separation and biological evaluation. Preparative medium-pressure liquid chromatographic (MPLC) separation for F3 (4.0 g) on a glass column of reverse-phase silica gel (LiChroprep^®^ RP-18, 25–50 μm; Merck, Darmstadt, Germany), using CH_3_CN–H_2_O (0.5:4.5 to 1.5:3.5, gradient, flow rate: 1 mL/min) as a solvent system yielded IR3G (1.4 g).

### 4.3. Determination of the Total Phenolic Content (TPC), Total Flavonoid Content (TFC), and Antioxidant Activity of DHE

TPC and TFC in DHE were estimated, as described previously [93], using Folin–Ciocalteu and AlCl_3_ reagents. The absorbance of the produced colored products was measured at 760 and 415 nm, respectively. The calibration curves of absorbance vs. concentration for the standard solutions of gallic acid and quercetin were used to quantify TPC and TFC content, respectively.

The antioxidant capacity of DHE was assessed by measuring the decrease of the absorption of 2,2-diphenyl-1-picrylhydrazyl (DPPH) radical solution at 517 nm or 2,2′-Azino-bis(3-ethylbenzthiazoline-6-sulfonic acid) (ABTS) radical solution at 734 nm after addition of DHE solution, as previously reported [93,94].

### 4.4. Crystallographic Study

The compound IR3G was obtained as single crystals by slow evaporation from MeOH solution of the pure compound at room temperature. Data were collected on a Bruker APEX-II D8 Venture area diffractometer, equipped with graphite monochromatic Cu Kα radiation, λ = 1.54178 Å at 293 (2) K. Cell refinement and data reduction were carried out by Bruker SAINT. SHELXT was used to solve the structure. The final refinement was carried out by full-matrix least-squares techniques with anisotropic thermal data for nonhydrogen atoms (Cambridge Crystallographic Data Center (CCDC 1545557) contains the supplementary crystallographic data for this compound and can be obtained free of charge via www.ccdc.cam.ac.uk/data_request/cif, accessed on 10 January 2022).

### 4.5. Bioassays

#### 4.5.1. In Vitro Anti-Inflammatory Assay

The murine macrophage RAW 264.7 cells were obtained from American Type Culture Collection (ATCC: No. TIB-71, Manassas, VA, USA) and cultured as described previously [95]. The cultures were maintained in a humidified atmosphere of 5% CO_2_ in air at 37 °C, and the trypsinized cells were subcultured overnight in different-sized plastic wells plates (Corning Inc., NY, USA). Inflammation in macrophage cells was made by incubating them in *Escherichia coli* lipopolysaccharide (LPS; Sigma-Aldrich, St. Louis, MO, USA)-containing medium (0.01 μg/mL) without the tested materials. For testing the anti-inflammatory activity, IR3G or DHE was added to the macrophage cells 5 min before challenge with LPS. The cells were then washed with ice-cold PBS, lysed in ice-cold lysis buffer, centrifuged at 20,000× *g* at 4 °C for 30 min, and the supernatants were kept for western blot analysis. Protein concentrations were estimated by using DC protein assay kit (Bio-Rad Laboratories, Hercules, CA, USA) modified from the method of Lowry et al. [96].

For western blotting, the samples containing equal quantities of proteins in sodium dodecyl sulfate (SDS) buffer (2% SDS, 2% 2-mercaptoethanol, 0.1% bromophenol blue, 10% glycerol, and 50 mM Tris-HCl of pH 7.2) were loaded on a 10% SDS–polyacrylamide gel, followed by electrophoresis at 150 V for 90 min. The separated proteins were transferred onto a 0.45 μm pore sized-Immobilon-P polyvinylidene difluoride (PVDF) membrane (Millipore Corp., Billerica, MA, USA) overnight at 4 °C in a transfer buffer. The resulting PVDF membranes were blocked with 5% skimmed milk powder in Tris-buffered saline containing 0.1% Tween (pH 7.4) and then incubated at 4 °C overnight with the 1ry antibodies: iNOS (Transduction Laboratories, San Diego, CA, USA), COX-2 (Cayman Chemicals, Ann Arbor, MI, USA), and β-actin (Sigma-Aldrich, St. Louis, MO, USA). The horseradish peroxidase-conjugated 2ry antibody was used for detection. The proteins were visualized, and their relative densitometric quantification was performed using chemiluminescence (Millipore Corp., Billerica, MA, USA) and LabWorks v6.2 software (UVP Inc., Upland, CA, USA), respectively. The anti-β-actin antibody was employed as a loading control.

#### 4.5.2. In Vitro Neuroprotective Assay

The human neuroblastoma SH-SY5Y cell line was obtained from American Tissue Culture Collection (Manassas, VA, USA) and cultured and used to test the neuroprotective activity described previously [36,97]. The cells were treated with the tested compound at concentrations of 0.0001, 0.001, 0.01, and 0.1 μM for one h and then exposed to 6-hydroxydopamine (6-OHDA, Sigma-Aldrich, MO, USA) at 20 μM. The SHSY5Y cells’ survival was determined after 18 h of incubation using Alamar blue assay. Relative protection (%) was calculated as (optical density (OD) of 6-OHDA + IR3G-treated cells − OD of 6-OHDA alone-treated cells)/(OD of normal control cells − OD of 6-OHDA alone-treated cells)) × 100.

#### 4.5.3. Locomotor Activity Assay in Zebrafish

The wild-type zebrafish (AB strain) was used. The embryos were collected after standard natural spawning and raised synchronously in Hank’s buffer (pH 7.4) at 28.5 °C. No additional care was required, as the embryos have nourishment from the attached yolk sac. The effect on locomotor activity was assayed in zebrafish according to the previous protocol [30]. Zebrafish larvae at two days post-fertilization (2 dpf) were challenged with 250 μM 6-OHDA (2 to 5 dpf) in the presence or absence of tested compound (9 h post-fertilization, hpf to 5 dpf) in a 24-well plate. The swimming behavior was monitored with an automated video tracking system (Singa Technology Co. Ltd., Taipei, Taiwan).

The experimental protocols of the in vivo study were approved by Animal Care and Use Committee of National Sun Yat-sen University (approval no. 10303).

### 4.6. In Silico Study

#### 4.6.1. Molecular Docking Study

The molecular docking study investigated how isorhamnetin 3-O-β-D-glucopyranoside (IR3G), in comparison with isorhamnetin (IR), interacts with the expected targets: acetylcholinesterase (AChE), monoamine oxidase-B (MAO-B), and brain-permeable Polo-like kinase-2 (PLK-2), separately. The docking analysis was carried out in a molecular operating environment (MOE), and target structures were obtained from the protein data bank in PDB file format (PDB ID: 4M0E, 6FVZ, and 4I5P, respectively). In the MOE, the IR3G and IR structures were converted to the MDB file format. For energy minimization, all docking settings were left at their default values. The scoring system was set up with London dG and (GBVI/WSA dG) as functions 1 and 2, respectively.

#### 4.6.2. Molecular Dynamics (MD) Simulation Study

MD simulation of IR3G or IR was carried out on AChE, MAO-B, and PLk2, separately. The docked structures of the protein in a complex with an inhibitor were used as a starting point for the computational simulations. For 20 ns, simulations in a periodic water box were performed using the Chemistry at Harvard Macromolecular Mechanics 36 (CHARMM36) force field and the Nanoscale Molecular Dynamics (NAMD) package version 2.13 [98,99]. The ligand force field was created using the CHARMM-GUI server [60]. The water box (containing 0.150 mM NaCl) was formed by gradually adding water in the positive and negative x, y, and z directions around the protein for 20 Å, resulting in a cuboidal box. The Lennard-Jones (LJ) cutoff distance was set at 12, while the switching distance was set at 10. The particle mesh Ewald (PME) approach was used to deal with long-range electrostatic interactions. One thousand minimization steps of a conjugate gradient method were used to prepare systems before they were put into production. The simulations were carried out in standard pressure and temperature (NPT) ensemble held constant at 300 K and 1 bar by means of a Langevin thermostat and a barostat. A number of analyses were carried out following the simulation, including root mean square deviation (RMSD), root mean square fluctuations (RMSF), and gyration radius [100].

### 4.7. Biphasic Qualitative Identification and Quantitative Estimation of IR3G in DHE by HPTLC Densitometry

An HPTLC method was developed and validated as described [101]. Normal and reverse-phase (NP and RP)-HPTLC plates (Merck) were used for qualitative identification and quantitative determination of the isolated pure IR3G in DHE. HPTLC system of CAMAG^®^ (CAMAG, Muttenz, Switzerland) composed of automatic TLC sampler (ATS-4), automatic development chamber (ADC-2), and TLC visualizer-2 with CCD camera were employed for band-wise application of IR3G and DHE to the NP- or RP-HPTLC plate, plate development, and plate scanning and documentation, respectively. The whole process of spotting, plate development, and analysis was controlled by CAMAG WinCATS software v.1.3.4. The results are presented in Table 3 and Figure 10 and Figure 11.

#### 4.7.1. HPTLC Instrumentation and Conditions

The HPTLC analysis IR3G in DHE was completed on glass HPTLC plates (20 × 10 cm), where the band of each track was 6 mm wide and 7.9 mm apart. Both marker (IR3G) and extract (DHE) were applied on the HPTLC at a rate of 160 nL s−1 (in spray mode) with the ATS-4 sampler’s microliter syringe. The plates were then developed in a presaturated twin-trough glass chamber with ideal saturation conditions (at 25 ± 2 °C and 60 ± 5% humidity). The developed HPTLC plates were dried, and IR3G bands were quantitatively analyzed based on the absorbance density at 254 nm. IR3G identification in DHE was based on Rf values in comparison with that of standard IR3G, which was further confirmed by the superimposed UV spectra measured from 200 to 400 nm (Figure 10).

#### 4.7.2. Preparation of Standard Stock Solutions and Calibration Curves

A stock solution of IR3G (1 mg mL^−1^) was prepared by dissolving in MeOH, which was further diluted with MeOH to afford seven concentrations of 10–70 μg mL^−1^ (10–70 ng μL^−1^). A volume of 10 μL from each dilution was applied on the HPTLC (*n* = 3) to give the linearity range of 100–700 ng band^−1^. A methanolic solution of DHE (0.3 g /100 mL MeOH) was prepared by sonication for 5 min, followed by passing through a Millipore filter. The clear solution was used for HPTLC analysis and spectrophotometric determination of total IR3G in DHE sample. The calibration curve for standard IR3G was prepared by applying the concentrations range of 100–600 ng IR3G per band against the absorption density at 254 nm. Two calibration curves for the standard IR3G solutions were synthesized according to the NP- and RP-HPTLC mode. The solvent systems EtOAc–CH_2_Cl_2_–HCOOH (9:1:1) and CH_3_CN–H_2_O (3:7) were used as mobile phases for the development of the NP- and RP-HPTLC, respectively.

#### 4.7.3. HPTLC Densitometric Quantitative Determination of IR3G in DHE and Method Validation

After application of the reference biomarker (IR3G) and mother extract (DHE) solutions on the NP- or RP-HPTLC plate, the percentages of IR3G in DHE solution were estimated by measuring the absorption density (based on peak area) of IR3G in both of the reference and the extract solutions at 254 nm using TLC visualizer-2. The percentage of IR3G in DHE was then calculated with the calibration curves mentioned in step 4.7.2. The *R_f_* values of IR3G (*n* = 3) in the NP- and RP-HPTLC systems are depicted in Figure 11. The HPTLC method was further validated by the determination of the limit of detection (LOD), the limit of quantification (LOQ), and the linearity range in both chromatographic systems.

### 4.8. Statistical Analysis

All biological data are represented as the mean ± standard error of the mean. Wherever applicable, data were analyzed by one-way analysis of variance followed by Dunnett’s test; *p*-value < 0.05 is considered statistically significant.

## 5. Conclusions

From the antioxidant, anti-inflammatory ethanolic extract of *D. harra* (DHE), isorhamnetin-3-O-β-D-glucoside (IR3G) was isolated and quantified by a biphasic HPTLC method as a major constituent (ca. 1.207–1.228% *w*/*w*). The underlying mechanism of IR3G in combatting oxidative stress-induced neurodegenerative conditions was studied. The mechanism of IR3G was partially proved by its structure–antioxidant relationship, anti-iNOS, anti-COX-2, and the protective activities shown against two Parkinson’s models of 6-OHDA-induced neurotoxicity in human dopaminergic neurons and locomotion deficit in zebrafish,. The results of molecular docking and MD simulation analyses further revealed the inhibitory potentiality of IR3G and its aglycone IR against the PD-related neurogenic targets (human MAO-B, AChE, and PLK2). The current study thus suggests IR3G as a promising natural therapeutic agent against PD and inflammatory conditions, such as RA. However, future research is required to experimentally evaluate the inhibitory activities against the three PD-linked pharmacological targets along with the pharmacokinetic properties of IR3G. A study of the effect of combination therapy of IR3G with levodopa (a standard PD medication) in the aim of improvement of clinical symptoms in PD patients should also be considered. This combination may be superior to levodopa monotherapy in terms of synergizing the pharmacodynamic effect and/or reducing the dose of levodopa.

## Figures and Tables

**Table 1:** Total phenol (TPC)/flavonoid (TFC) contents and antioxidant activity of DHE.

Index		Value
Phenol content ^a^	(mg GAE/g)	80.43 ± 0.01
Flavonoid content ^b^	(mg QUE/g)	54.26 ± 0.03
Antioxidant activity	(IC_50_ μg/mL) ^c^	247.4 [5.9] ^e^
	(IC_50_ μg/mL) ^d^	203.7 [6.8] ^f^

Indices were measured using ^a^ Folin–Ciocalteau and ^b^ AlCl3 reagent methods and ^c^ DPPH and ^d^ ABTS radical scavenging assays. Values obtained by reference antioxidants: ^e^ quercetin and ^f^ ascorbic acid. GAE = gallic acid equivalent and QUE = quercetin equivalent.

**Table 2:** Docking energy and interaction of IR3G and IR with target proteins (4M0E, 6FVZ, and 4I5P).

Compound Code	Target (PDB Code)	Ligand	Receptor	Interaction	Distance (Å)	E (kcal/mol)	Docking Score (kcal/mol)
IR3G	AChE (4M0E)	O 3	OD2 ASP74 (A)	H-donor	2.10	−1.9	−7.7045
		O 4	OH TYR124 (A)	H-acceptor	2.18	−0.1	
		O 6	N PHE295 (A)	H-acceptor	2.29	−0.5	
		C 17	6-ring TRP286 (A)	H-pi	2.61	−0.6	
	MAO-B (6FVZ)	O 3	O PRO102 (A)	H-donor	2.72	−1.8	−5.863
		O 6	O LEU164 (A)	H-donor	3.52	−0.2	
		C 29	SG CYS172 (A)	H-donor	3.06	−0.3	
		O 8	CD1 PHE168 (A)	H-acceptor	3.38	0.6	
		6-ring	CA ILE199 (A)	pi-H	4.08	−0.6	
		6-ring	CA ILE199 (A)	pi-H	4.94	−0.2	
	PLK-2 (4I5P)	O 3	OD1 ASP223 (A)	H-donor	3.23	−1.3	−7.7340
		O 4	OD1 ASN210 (A)	H-donor	3.14	−1.2	
		O 5	SG CYS96 (A)	H-donor	4.29	−1.0	
		O 6	O GLY209 (A)	H-donor	3.07	−1.1	
		6-ring	CB LEU88 (A)	pi-H	4.11	−0.8	
		6-ring	CB LEU88 (A)	pi-H	4.61	−0.4	
		6-ring	CD1 LEU88 (A)	pi-H	4.51	−0.5	
		6-ring	CD2 LEU88 (A)	pi-H	4.5	−0.5	
		6-ring	CG ARG165 (A)	pi-H	4.99	−0.3	
IR	AChE (4M0E)	O 5	N ARG296 (A)	H-acceptor	3.31	−2.2	−6.8919
		C 23	5-ring TRP286 (A)	H-pi	4.01	−0.5	
		6-ring	CE1 PHE297 (A)	pi-H	4.86	−0.3	
		6-ring	6-ring TRP286 (A)	pi-pi	3.79	−0.1	
	MAO-B (6FVZ)	O 6	O PRO102 (A)	H-donor	1.85	−3.2	−7.5290
		O 5	CA PHE168 (A)	H-acceptor	2.11	−1.1	
		6-ring	CA CYS172 (A)	pi-H	3.72	−0.4	
		6-ring	CB ILE199 (A)	pi-H	3.78	−0.4	
	PLK-2 (4I5P)	O 6	O LEU88 (A)	H-donor	3.09	−1.5	−6.4320
		C 21	SG CYS96 (A)	H-donor	4.04	−0.5	
		O 2	N CYS162 (A)	H-acceptor	3.11	−0.4	
		O 5	N CYS162 (A)	H-acceptor	2.97	−1.3	
		6-ring	CB LEU88 (A)	pi-H	4.69	−0.3	
		6-ring	CB LEU88 (A)	pi-H	4.08	−0.4	
		6-ring	CD1 LEU88 (A)	pi-H	4.08	−0.8	
		6-ring	CD1 LEU88 (A)	pi-H	4.47	−0.5	
		6-ring	CD2 LEU88 (A)	pi-H	4.04	−0.7	
		6-ring	CG ARG165 (A)	pi-H	4.75	−0.4	

IR3G: isorhamnetin-3-O-β-D-glucoside; AChE: Acetylcholine esterase; MAO-B: Monoamine oxidase type B; PLK-2: Polo-like kinase-2.

**Table 3:** Linear regression data with LOD and LOQ for IR3G in NP- and RP-HPTLC (*n* = 3).

	NP-HPTLC	RP-HPTLC
Linearity range (ng/band)	100–400	100–500
Regression equation	Y = 7.933 + 0.4428X	Y= 0.816 + 0.346X
Correlation coefficient (r^2^)	0.99296	0.99018
Slope	0.4428	0.346
Standard deviation (Sdv)	5.43%	7.09%
Limit of detection (LOD; ng)	40.47	67.6
Limit of quantification (LOQ; ng)	122.63	204.91

## Data Availability

The data are included within the article or Supplementary Materials. CCDC 1545557 contains the supplementary crystallographic data for the isolated compound that can be obtained free of charge from the Cambridge Crystallographic Data Centre via http://www.ccdc.cam.ac.uk/data_request/cif (accessed on 10 January 2022).

## Supplementary Materials

The following are available online at https://www.mdpi.com/article/10.3390/plants11050648/s1, Figure S1: TLCs and a bioautographic TLC of F3; Figure S2: The 2D structures of IR3G and IR; Table S1: Experimental details of the X-ray diffraction analysis; Table S2: Selected geometric parameters (Å, °); Table S3: Hydrogen-bond geometry (Å, °); Table S4. Redocking energy and interaction of ligands (1YL, E8Z, and 1D1) with target proteins 4M0E, 6FVZ, and 4I5P, respectively. References [102,103,104] are cited in the supplementary materials.
