# Supplementary material for: A Major Diplotaxis harra-Derived Bioflavonoid Glycoside as a Protective Agent against Chemically Induced Neurotoxicity and Parkinson’s Models; In Silico Target Prediction; and Biphasic HPTLC-Based Quantification
Source: Plants (Basel). 2022 Feb 27;11(5):648. doi: 10.3390/plants11050648 (PMC8912516; doi:10.3390/plants11050648)
Supplement: Supplementary file 1 [file plants-11-00648-s001.zip › plants-1619370-supplementary.pdf]

# A Major Diplotaxis harra-Derived Bioflavonoid Glycoside as a Protective Agent against Chemically Induced Neurotoxicity and Parkinson's Models; In Silico Target Prediction; and Biphasic HPTLC-Based Quantification

**Atallah F. Ahmed [1,2,*], Zhi-Hong Wen [3], Ahmed H. Bakhiet [4], Omer A. Basudan [1], Hazem A. Ghabbour [5], Abdullah Al-Ahmari [1], Chien-Wei Feng [6,7]**

1. Department of Pharmacognosy, College of Pharmacy, King Saud University, P.O. Box 2457, Riyadh 11451, Saudi Arabia
2. Department of Pharmacognosy, Faculty of Pharmacy, Mansoura University, El-Mansoura 35516, Egypt
3. Department of Marine Biotechnology and Resources, National Sun Yat-sen University, Kaohsiung 804, Taiwan
4. Department of Pharmaceutical Chemistry, College of Pharmacy, King Saud University, P.O. Box 2457, Riyadh 11451, Saudi Arabia
5. Department of Medicinal Chemistry, Faculty of Pharmacy, University of Mansoura, Mansoura 35516, Egypt
6. Department of Obstetrics and Gynecology, Kaohsiung Medical University Hospital, Kaohsiung 807377, Taiwan
7. Center for Cancer Research, Kaohsiung Medical University, Kaohsiung 807377, Taiwan

.

Author to whom correspondence should be addressed; E-Mail: afahmed@ksu.edu.sa; Tel.: +966-114-677-264; Fax: +966-114-677-245.

*1. TLCs and a bioautographic TLC of F3*

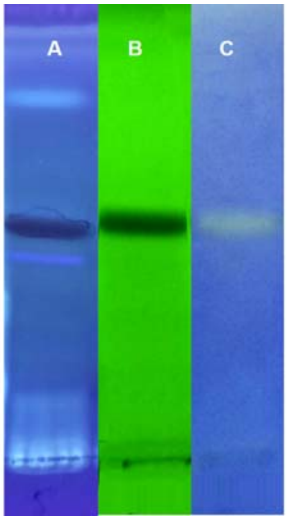


**Figure S1.** Silica gel TLC chromatograms of EtOAc fraction (F3) as visualized under UV light at 366 nm (A), at 254 nm (B) and after exposure to 0.5 % w/v methanolic DPPH spray reagent under white light (C). Solvent system: EtOAc – MeOH – $H_2O$ (10:1.5:1).

*2. X ray diffraction analysis*

The isolated Isorhamnetin-3-O-β-D-glucoside (IR3G) was obtained as single crystals by slow evaporation from ethanol solution of the pure compound at room temperature. Data were collected on a Bruker APEX-II D8 Venture area diffractometer, equipped with graphite monochromatic Cu $K\alpha$ radiation, $\lambda$ = 1.54178 Å at 293 Cell refinement and data reduction were carried out by Bruker SAINT. The SHELXT [1, 2] was used to solve structure. The final refinement was carried out by full-matrix least-squares techniques with anisotropic thermal data for nonhydrogen atoms on $F$. CCDC 1545557 contains the supplementary crystallographic data for this compound can be obtained free of charge from the Cambridge Crystallographic Data Centre *via* www.ccdc.cam.ac.uk/data_request/cif. The molecular structure of IR3G, $C_{22}H_{22}O_{12}\cdot 2(H_2O)$, was crystallized in the Monoclinic, $C2$, $a$ = 24.2571 (7) Å, $b$ = 11.5984 (3) Å, $c$ = 9.3161 (3) Å, $\beta$ = 107.456 (1)°, $V$ = 2500.32 (13) Å$^3$, Z = 4.

The crystallographic data and refinement information are summarized in Table S1. The selected bond lengths and bond angles are listed in Table S2. The asymmetric unit is containing of one independent molecule as shown in Figure 1. All the bond lengths and angles are in normal ranges [3]. In the crystal packing, molecules are linked *via* intermolecular hydrogen bonds (Figure 1 and Table S3).

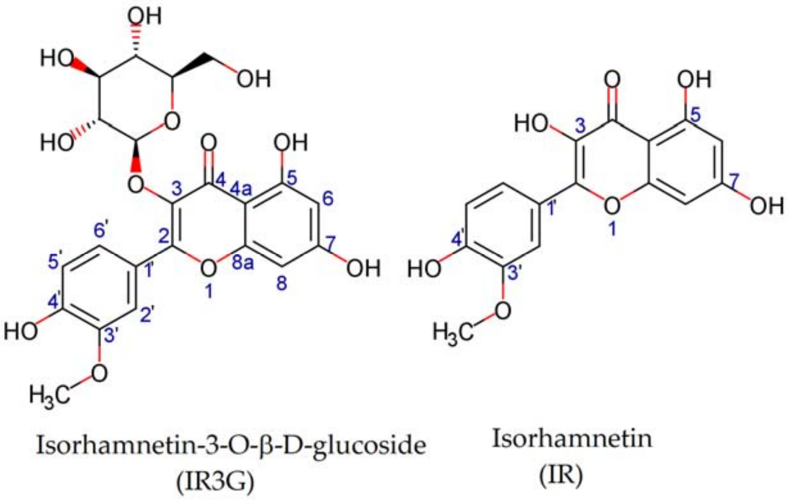


**Figure S2**. The 2D **s**tructures of IR3G and IR

**Table S1**. Experimental details of the X ray diffraction analysis

| | |
|---|---|
| **Crystal data** | |
| Chemical formula | $C_{22}H_{22}O_{14}$ |
| Mr | 510.40 |
| Crystal system, space group | Monoclinic, *C*2 |
| Temperature (K) | 293 |
| *a*, *b*, *c* (Å) | 24.2571 (7), 11.5984 (3), 9.3161 (3) |
| *β* (°) | 107.456 (1) |
| V (Å$^3$) | 2500.32 (13) |
| Z | 4 |
| Radiation type | Cu *Kα* |
| Crystal size (mm) | 0.54 × 0.25 × 0.12 |
| **Data collection** | |
| Diffractometer | Bruker APEX-II D8 venture diffractometer |
| Absorption correction | Multi-scan SADABS Bruker 2014 |
| Tmin, Tmax | 0.774, 0.810 |
| No. of measured, independent and observed [I > 2σ(I)] reflections | 32484, 4933, 4596 |
| $R_{int}$ | 0.056 |
| **Refinement** | |
| R[$F^2 > 2\sigma(F^2)$], wR( $F^2$), S | 0.062, 0.193, 1.11 |
| No. of reflections | 4933 |
| No. of parameters | 343 |
| No. of restraints | 1 |
| H-atom treatment | H atoms treated by a mixture of independent and constrained refinement |
| $\Delta\rho_{max}$, $\Delta\rho_{min}$ (e Å$^{-3}$) | 0.86, −0.27 |

**Table S2.** Selected geometric parameters (Å, °)

| | | | |
|---|---|---|---|
| O1—C1 | 1.362 (3) | O6—C22 | 1.425 (5) |
| O1—C9 | 1.355 (3) | O7—C13 | 1.354 (4) |
| O2—C3 | 1.351 (4) | O8—C17 | 1.406 (4) |
| O3—C5 | 1.346 (4) | O9—C18 | 1.426 (4) |
| O4—C7 | 1.255 (3) | O10—C19 | 1.426 (4) |
| O5—C8 | 1.376 (3) | O11—C21 | 1.398 (6) |
| O5—C16 | 1.419 (3) | O12—C16 | 1.418 (3) |
| O6—C12 | 1.349 (4) | O12—C20 | 1.445 (4) |
| C1—O1—C9 | 122.3 (2) | O6—C12—C13 | 115.3 (2) |
| C8—O5—C16 | 118.1 (2) | O6—C12—C11 | 124.4 (2) |
| C12—O6—C22 | 117.9 (3) | O7—C13—C12 | 117.0 (3) |
| C16—O12—C20 | 112.0 (2) | O7—C13—C14 | 123.5 (2) |
| O1—C1—C6 | 120.0 (3) | O5—C16—O12 | 106.68 (19) |
| O1—C1—C2 | 116.8 (3) | O5—C16—C17 | 105.0 (2) |
| O2—C3—C2 | 121.6 (3) | O12—C16—C17 | 111.6 (2) |
| O2—C3—C4 | 116.6 (3) | O8—C17—C16 | 110.9 (2) |
| O3—C5—C6 | 120.3 (3) | O8—C17—C18 | 107.3 (2) |
| O3—C5—C4 | 119.2 (3) | O9—C18—C19 | 111.2 (2) |
| O4—C7—C8 | 122.9 (2) | O9—C18—C17 | 111.0 (2) |
| O4—C7—C6 | 121.4 (3) | O10—C19—C18 | 109.7 (2) |
| O5—C8—C9 | 120.4 (2) | O10—C19—C20 | 107.6 (3) |
| O5—C8—C7 | 117.6 (2) | O12—C20—C19 | 109.0 (3) |
| O1—C9—C10 | 110.2 (2) | O12—C20—C21 | 107.5 (3) |
| O1—C9—C8 | 120.5 (2) | O11—C21—C20 | 112.8 (3) |

**Table S3.** Hydrogen-bond geometry (Å, °)

| *D*—H···*A* | *D*—H | H···*A* | *D*···*A* | *D*—H···*A* |
|---|---|---|---|---|
| O8—H1O8···O4$^{i}$ | 1.05 (6) | 1.83 (6) | 2.850 (3) | 163 (5) |
| O9—H1O9···O2W | 0.72 (4) | 2.01 (5) | 2.710 (4) | 167 (5) |
| O2—H2B···O1W | 0.8200 | 1.8000 | 2.581 (7) | 158.00 |
| O7—H1O7···O9$^{ii}$ | 0.76 (5) | 1.99 (5) | 2.701 (3) | 157 (5) |
| O3—H3A···O4 | 0.8200 | 1.8800 | 2.607 (3) | 147.00 |
| O2W—H4OW···O6$^{iii}$ | 0.70 (7) | 2.19 (7) | 2.819 (5) | 151 (7) |
| O2W—H4OW···O7$^{iii}$ | 0.70 (7) | 2.58 (7) | 3.160 (5) | 142 (7) |
| O10—H10A···O11$^{iv}$ | 0.8200 | 1.9100 | 2.717 (4) | 166.00 |
| O11—H11O···O10$^{v}$ | 0.50 (5) | 2.26 (5) | 2.717 (4) | 152 (6) |
| O2W—H23W···O3$^{i}$ | 0.74 (7) | 2.27 (7) | 2.972 (5) | 159 (6) |
| C11—H11A···O12 | 0.9300 | 2.5000 | 3.259 (3) | 139.00 |

Symmetry codes: (i) −*x*+1, *y*, −*z*+1; (ii) *x*, *y*−1, *z*; (iii) −*x*+1/2, *y*+1/2, −*z*; (iv) −*x*+1/2, *y*+1/2, −*z*+1; (v) −*x*+1/2, *y*−1/2, −*z*+1.

**Table S4.** Redocking energy and interaction of ligands (1YL, E8Z, and 1D1) with target proteins (4M0E, 6FVZ, and 4I5P), respectively.

| Compound | Target (PDB code) | Ligand | Receptor | Interaction | Distance | E (kcal/mol) | Docking score (kcal/mol) |
|---|---|---|---|---|---|---|---|
| 1YL (PDB code) | AChE (4M0E) | C18 1 | 5-ring TRP286 (A) | H-pi | 3.31 | –0.3 | –6.4471 |
| | | 6-ring | 6-ring TRP286 (A) | pi-pi | 3.86 | –0.1 | |
| | | 6-ring | 6-ring TRP286 (A) | pi-pi | 3.62 | –0.1 | |
| E8Z (PDB code) | MAO-B (6FVZ) | CAK 14 | SG CYS172 (A) | H-donor | 3.22 | –0.6 | –7.5971 |
| | | 6-ring | CA CYS172 (A) | pi-H | 3.49 | –1.1 | |
| | | 6-ring | CB ILE199 (A) | pi-H | 2.70 | –0.6 | |
| 1D1 (PDB code) | PLK-2 (4I5P) | C8 9 | O GLU160 (A) | H-donor | 3.16 | –1 | –8.5290 |
| | | O23 1 | O HOH519 (A) | H-acceptor | 2.84 | –2.4 | |
| | | O23 1 | O HOH617 (A) | H-acceptor | 3.07 | -0.9 | |
| | | N7 11 | N CYS162 (A) | H-acceptor | 2.89 | –5.3 | |
| | | 5-ring | CD1 LEU88 (A) | pi-H | 4.16 | –0.7 | |

IR3G: Isorhamnetin-3-O-β-D-glucoside; AChE: Acetylcholine esterase; MAO-B: Monoamine oxidase type B; PLK-2: Polo-like kinase-2; 1YL: Dihydrotanshinone I; E8Z: N-(3,4-dimethylphenyl)-4-oxidanylidene-chromene-3-carboxamide; 1D1: (7R)-8-cyclopentyl-7-ethyl-5-methyl-2-(1H-pyrrol-2-yl)-7,8-dihydropteridin-6(5H)-one.

**References:**


1. Sheldrick, G. M., A short history of SHELX. *Acta Crystallogr. A* **2008,** 64, (Pt 1), 112-122.
2. Sheldrick, G. M., SHELXTL-PC (Version 5.1), Siemens analytical. instruments, Inc., Madison, WI, 1997.
3. Allen, F. H.; Kennard, O.; Watson, D. G.; Brammer, L ;.Orpen, A. G.; Taylor, R., Tables of Bond Lengths determined by X-Ray and Neutron Diffraction. Part. 1. Bond Lengths in Organic Compounds. *J. Chem. Soc., Chem. Soc. Perkins Trans. 2* **1987**, S1–S19.
